# A comparison of social drivers of health identification and intervention rates by sex among patients receiving primary care

**DOI:** 10.1186/s13293-025-00738-z

**Published:** 2025-07-25

**Authors:** Leah A. Holcomb, Elizabeth Crabtree Killen, Kelsey R. Ryan, Aimee L. McRae-Clark, Stacey Seipel, Rita Aidoo, Constance Guille

**Affiliations:** 1 Department of Psychiatry and Behavioral Sciences, Medical University of South Carolina, Charleston, SC USA; 2 Department of Public Health Sciences, Medical University of South Carolina, Charleston, SC USA; 3 Population Health, Medical University Hospital Authority Benefits Department, Medical University of South Carolina, Charleston, SC USA; 4 Department of Obstetrics and Gynecology, Medical University of South Carolina, Charleston, SC USA

**Keywords:** Gender, Social determinants of health, Social drivers of health, Sex

## Abstract

**Background:**

Social drivers of health (SDOH) significantly influence health behaviors and outcomes, yet sex-based disparities in these domains remain underexplored. Identifying these differences is essential for guiding equitable, evidence-based interventions.

**Methods:**

We analyzed electronic health record (EHR) data from all patients with a documented male or female sex who had a primary care visit or inpatient stay at the Medical University of South Carolina (MUSC) between January 1, 2023, and December 31, 2024 (*n* = 493,920). SDOH screening responses were categorized as “affirmative” (at risk) or “negative” (not at risk) across 17 predefined domains using Epic’s logic-based risk classification. Descriptive statistics were calculated, and z-tests for proportions were used to assess sex-based differences. Race and ethnicity were included as descriptive variables; no inferential tests by race/ethnicity were conducted.

**Results:**

Females were significantly more likely to report financial strain (7.96%), food insecurity (4.44%), housing instability (3.72%), intimate partner violence (2.03%), transportation barriers (2.20%), depression (3.93%), and stress (14.10%). Despite these risks, females also reported higher rates of protective behaviors such as physical activity (74.2%) and social connectedness (14.22%). In contrast, males had higher rates of alcohol use (4.67%), tobacco use (35.6%), and adolescent substance use (2.14%). Notably, White/Caucasian males reported the highest alcohol use (6.23%), and both White and Black males reported the highest tobacco use (42%).

**Conclusions:**

Sex-based disparities in SDOH reflect broader structural and social inequities. Health systems should implement routine, EHR-integrated SDOH screening and use this data to inform tailored, gender-responsive interventions—such as increasing access to mental health support for women and addressing substance use among men—while also considering how intersecting factors like race, income, and caregiving burden compound these risks.

## Plain language summary

Social Drivers of Health (SDOH), such as access to housing, food, and financial resources, can impact overall well-being and health outcomes. This study examined gender differences in SDOH by analyzing responses across different domains among a sample of individuals who received primary care at a regional health system in the Southeastern United States. While males and females had similar overall rates of positive responses (69% for both groups), there were significant differences when looking at individual domains. These findings suggest that males and females may experience different challenges related to SDOH, which could impact their ability to access resources and support services. Understanding these differences can help shape policies and programs that better address the unique needs of both men and women, ultimately improving health equity.

## Highlights

- This study examined sex differences in key social drivers of health (SDOH) needs among a large sample of individuals receiving care at an academic medical center in the southeastern U.S.
- Both males and females had nearly identical overall rates of reported unmet SDOH needs, with approximately 69% reporting at least one need.
- Despite similar overall rates, significant sex differences emerged across specific SDOH domains, highlighting distinct challenges faced by men and women.
- Identifying these sex-specific differences in SDOH needs can help tailor interventions and policies to better support individuals in accessing necessary resources and services.

## Background

The World Health Organization (WHO) conceptualizes gender as a core component of the social drivers of health (SDOH) framework, which highlights how nonmedical factors—such as economic stability, healthcare access, education, and community environments—influence health outcomes [1, 2]. Gender intersects with these determinants through differential risk of health outcomes, unequal access to care, and systemic bias [3]. Because gender is socially constructed, its impact on health varies across contexts, with norms and expectations shaping individuals’ opportunities and constraints. For example, women often face distinct disparities—including reduced economic security, barriers to healthcare access, and higher rates of violence—as well as historic underrepresentation in biomedical research, all of which contribute to inequitable health outcomes [4]. Sex and gender are distinct but interrelated concepts that both influence health. Sex refers to biological attributes such as chromosomes, hormone levels, and reproductive anatomy, whereas gender is a social construct encompassing roles, behaviors, and identities shaped by cultural and societal norms. Within the framework of SDOH, gender operates as a key determinant that influences health through its interaction with other nonmedical factors such as income, education, and access to care [5]. In this study, we use the term sex rather than gender, as the data collected and analyzed were based on sex as documented in the electronic health record (EHR). While gender is a fundamental determinant of health—shaping individuals’ experiences and outcomes through its interaction with social, economic, and structural factors—our analysis is limited to sex as recorded within the EHR, and we refer to results accordingly.

Although women have achieved higher levels of education over time—a factor typically associated with increased earning potential—gender-based income disparities persist, limiting women’s economic stability [6]. These inequities have significant implications for health, as women are more likely to report lower income, screen positive for depression, and be unmarried, all of which are linked to poorer health outcomes over time [7]. Economic disadvantage is further compounded by social factors such as limited spousal or family support, inadequate childcare, and lack of reliable transportation, which contribute to unmet social needs and create barriers to accessing healthcare [8]. For low-income women in particular, caregiving responsibilities often force trade-offs between work, healthcare, and family obligations, increasing financial strain, mental health challenges, and risk for chronic disease [9].

Gender inequities also permeate the healthcare system. Women face stigmatization of health concerns and are more likely to be misdiagnosed, undertreated for conditions like heart disease, or receive inadequate pain management compared to men. Gender bias in clinical perception, particularly around pain, can lead to discounting or minimizing women’s symptoms, which can result in delays in diagnosis, insufficient pain management, and overall inadequate treatment. These patterns contribute to broader health disparities by reducing the quality and effectiveness of care women receive across a range of conditions [10–13]. Financial barriers compound these issues; women are more likely to delay or forgo care due to cost and report unmet healthcare needs even when controlling for health status [14–16].

SDOH, including income, employment, caregiving, and support, play varied roles in health outcomes for women and men [17]. While behavioral risk factors like smoking and alcohol use are more predictive for men, body weight and physical inactivity are more influential for women [18, 19]. Integrating both structural and behavioral determinants into health models is critical for understanding and addressing gender-based disparities. For example, incorporating SDOH into risk models improved cardiovascular outcome predictions, especially among non-Hispanic Black women with breast cancer [20].

From 1999 to 2018, barriers to timely medical care increased across all population groups in the United States, with widening disparities among racial and ethnic minorities [21]. These challenges reflect broader inequities in SDOH, which are often more prevalent among historically marginalized communities [22]. As a result, these groups frequently experience greater unmet social needs that negatively impact health and access to care. By 2022, life expectancy remained lowest among American Indian and Alaska Native (67.9 years) and Black individuals (72.8 years), compared to White individuals (77.5 years), with AIAN, Hispanic, and Black populations also experiencing steeper declines since 2019 [23]. These patterns underscore the compounding effects of structural racism, implicit bias, and social disadvantage on health outcomes, highlighting the need for more equitable, upstream approaches to care.

The COVID-19 pandemic further exposed gender disparities, with women—particularly from marginalized groups—experiencing disproportionate job loss, housing instability, and barriers to care [22]. These compounded stressors reinforce the urgency of addressing gender-specific SDOH to improve long-term health equity. Given well-documented gender-based disparities across economic, healthcare, and social domains, this study aimed to explore whether sex-based differences in SDOH needs were present among adult patients receiving primary care at a large academic medical center. We hypothesized that women would report a greater number of unmet SDOH needs compared to men, reflecting broader structural inequities that disproportionately affect women’s health and access to care.

## Methods

### Study setting

The Medical University of South Carolina (MUSC) has implemented a comprehensive, system-wide initiative to address SDOH through screening, documentation, and resource connection. Since 2021, this multidisciplinary approach has focused on: (1) integrating evidence-based SDOH screening tools into the electronic health record (EHR) to systematically identify and document SDOH risks, and (2) developing efficient workflows to connect patients with community resources. The initiative includes a digital community resource library containing over 1,600 community-based organizations across the state, enabling care teams to provide targeted referrals. To ensure a closed-loop referral process, MUSC employs a team of Community Health Workers (CHWs) who provide longitudinal, community-based social care support. CHWs receive referrals through an automated EHR-based system, triggered by SDOH screening responses collected via the patient portal or by front-line care team members. CHWs engage with patients through electronic communication, telephone outreach, and in-person visits to address identified social needs. SDOH program data are continuously monitored at the clinic/unit, division, and institutional levels through dashboards and standard reporting tools within the EHR. These data facilitate ongoing analysis to identify trends and inform continuous quality improvement efforts aimed at enhancing social care integration within the health system.

### Data extraction and analysis

A total of 494,067 patients who had either a primary care or inpatient visit at the Medical University of South Carolina (MUSC) between January 1, 2023, and December 31, 2024, were included in the initial dataset. Patients with an unknown or undetermined sex (*n* = 147) were excluded from the analysis, resulting in a final sample of 493,920 patients, comprising 290,958 females (58.9%) and 202,962 males (41.1%). A primary care visit was defined as an encounter within a department classified as “Primary Care” or “Family Medicine,” with an appointment status recorded as “completed” or “arrived.” For missing data, defined as instances where a patient declined to respond to a specific screening question, the patient was excluded from the analysis for that corresponding SDOH domain.

In this study, we use the term “sex” rather than “gender,” as the data collected and analyzed were based on sex as documented in the electronic health record (EHR). While gender is an important construct in understanding health disparities and social drivers of health, our analysis is limited to sex as recorded within the EHR, and we refer to results accordingly.

Descriptive statistics were calculated for the entire sample, and a difference of proportions test (z-test) was conducted to assess sex-based disparities in SDOH responses. The analysis evaluated patient responses to social drivers of health (SDOH) screening questions, categorizing responses as either affirmative (Yes = at risk) or negative (No = not at risk). Risk classification within each domain was based on the standardized logic outlined in Epic’s Social Drivers of Health Domain Questions, Answers, and Risk Classification Logic found in the Epic User Guide. This guide provides predefined criteria and algorithms used within the electronic health record (EHR) to group responses into specific SDOH domains, including intimate partner violence, social connections, financial resource strain, depression, stress, physical activity, food insecurity, transportation needs, housing stability, utility security, and personal safety. Patient responses were identified through EHRs and aggregated into predefined domains. Each domain was comprised of one or more questions, and embedded evaluation logic determined whether a need was flagged as affirmative. Additionally, age was categorized into 10-year increments for analysis, and results were consistent across these age groups. For missing data (i.e. when a patient refused to response), a patient is removed from the analysis for the domain of concern. All analyses were conducted in Microsoft Excel.

## Results

### Study population

A total of 494,067 patients who had either a primary care or inpatient visit at the Medical University of South Carolina (MUSC) between January 1, 2023, and December 31, 2024, were included in the initial dataset. After excluding 147 patients with unknown or indeterminate sex, the final analytic sample comprised 290,958 females (58.9%) and 202,962 males (41.1%).

### Demographic characteristics

Regarding racial and ethnic distribution (Table 1), among female patients, the largest group identified as White or Caucasian (*n* = 173,593, 59.7%), followed by Black or African American (*n* = 96,494, 33.2%). Among male patients, the largest group also identified as White or Caucasian (*n* = 130,228, 64.2%), followed by Black or African American (*n* = 56,499, 27.8%). Smaller racial and ethnic categories included American Indian or Alaska Native (Female: *n* = 865; Male: *n* = 620), Asian Indian (Female: *n* = 18; Male: *n* = 12), and Other Pacific Islander (Female: *n* = 296; Male: *n* = 227). A substantial proportion of patients either declined to respond (Female: *n* = 1,430; Male: *n* = 1,115) or were classified as “Unknown” (Female: *n* = 2,105; Male: *n* = 2,399). The study population included both children and adults, with 88% of patients between 18 and 64 years of age. The mean age was 50.6 years. Age distributions across racial and ethnic subgroups were generally similar, except for the “Other” racial category, which trended younger. Controlling for age did not impact the results.

Table 1 provides frequencies for all racial groups included in the study. 

**Table 1:** Patient counts by sex

Race	Female Patients		Male Patients	
	*N*	%	*N*	%
White or Caucasian	173,593	(59.66)	130,228	(64.16)
Black or African American	96,494	(33.16)	56,499	(27.84)
Other	11,997	(4.12)	8,757	(4.31)
Other Asian	3,491	(1.20)	2,231	(1.10)
Unknown	2,105	(0.72)	2,399	(1.18)
Patient Refused	1,430	(0.49)	1,115	(0.55)
American Indian or Alaska Native	865	(0.30)	620	(0.31)
Left Empty/Blank	617	(0.21)	838	(0.41)
Other Pacific Islander	296	(0.10)	227	(0.11)
Asian Indian	18	(< 0.01)	12	(< 0.01)

Table 2 provides overall percentages and key percent differences in SDOH.

**Table 2:** Social determinant of health domains

Domains	Female % Affirmative	Male % Affirmative	Female - Male Difference
Intimate Partner Violence	0.29%	0.20%	0.09%***
Social Connections	12.76%	11.21%	1.55%***
Alcohol Use	3.06%	5.39%	-2.33%***
Tobacco Use	28.99%	40.42%	-11.44%***
Financial Resource Strain	6.01%	4.55%	1.45%***
Depression	2.65%	1.67%	0.98%***
Stress	11.18%	7.30%	3.88%***
Physical Activity	24.30%	19.35%	4.96%***
Food Insecurity	3.50%	2.38%	1.11%***
Transportation Needs	1.60%	1.25%	0.35%***
Housing Stability	2.86%	2.03%	0.83%***
Health Literacy	1.11%	1.39%	-0.27%***
Utilities	0.53%	0.48%	0.05%*
Personal Safety	0.95%	0.44%	0.50%***
Adolescent Substance Use	2.63%	4.64%	-2.01%***
Adolescent Socialization	0.04%	0.05%	-0.01%

*p =.05, ***p =.001

### Social drivers of health analysis

SDOH data were analyzed based on patient responses to provider-administered screening questions, which assessed risk across multiple domains: intimate partner violence, social connections, alcohol use, tobacco use, financial resource strain, depression, stress, physical activity, food insecurity, transportation needs, housing stability, health literacy, utility security, personal safety, adolescent substance use, and adolescent socialization. Responses were categorized as “Yes” (affirmative/at risk) or “No” (negative/not at risk). Statistically significant differences in SDOH measures were observed between male and female patients.

Female patients reported higher rates of affirmative responses for intimate partner violence, social connections, financial resource strain, depression, stress, physical activity, food insecurity, housing stability, and personal safety. In contrast, male patients had higher rates of affirmative responses for alcohol use, tobacco use, health literacy, and adolescent substance use.

### Race and sex differences in SDOH responses

Further analysis revealed that American Indian or Alaska Native females had the highest mean number of affirmative SDOH responses, i.e. the average number of “at risk” designations per person with 1.19 per person, compared to the overall study population (1.02 per person), a statistically significant difference (*p* <.05). No other racial/sex subgroup exceeded an average of 1.06 affirmative responses per person. Regarding alcohol use, White/Caucasian individuals had the highest overall proportion of affirmative responses (4.82%), with White/Caucasian males reporting the highest subgroup rate (6.23%). Tobacco use was most prevalent among White/Caucasian and Black/African American males (42% each). Adolescent substance use was highest among White/Caucasian males (5.35%).

Among females, American Indian or Alaska Native individuals reported the highest levels of depression (3.93%), stress (14.10%), food insecurity (4.44%), and social connections (14.22%). Black/African American females reported the highest prevalence of financial resource strain (7.96%), while White/Caucasian females had high rates of depression (2.88%) and stress (12.63%). Physical activity deficits were most pronounced among Black/African American females (27.76%).

## Discussion

### Social and economic adversity among females

This study identified significant associations between sex and social determinants of health (SDOH), with females experiencing a disproportionately high burden of social and economic adversity. Although the overall proportion of affirmative SDOH responses was similar between sexes (approximately 69%), females reported significantly higher rates in nine of the eleven domains examined, including financial resource strain, food insecurity, housing instability, intimate partner violence, and personal safety. These factors are well-established contributors to poor mental and physical health outcomes [23–25]. Such disparities reflect systemic economic inequities, constrained healthcare access, and increased exposure to violence that women face, amplifying chronic stress and vulnerability to adverse health outcomes [4]. The persistence of these disparities underscores the need for healthcare and socioeconomic interventions that are specifically tailored to address structural factors shaping women’s health experiences.

### Protective health behaviors and the paradox of persisting mental health challenges

Interestingly, females also demonstrated higher engagement in protective behaviors such as physical activity and social connectedness. This pattern is consistent with research suggesting women tend to have greater health awareness, stronger social role expectations around relationships, and a greater propensity to seek emotional support during stress [26]. Physical activity is known to reduce cardiovascular risk, regulate stress hormones, and decrease depressive symptoms [27, 28]. Despite these protective behaviors, females reported higher rates of depression (3.93%) and stress (14.10%). This paradox suggests that while individual-level health behaviors are important, they cannot fully counteract the negative impacts of structural inequities. Chronic exposure to financial hardship, interpersonal violence (IPV), and limited access to resources, factors disproportionately experienced by women in this sample, likely override these benefits, perpetuating mental health disparities [3, 4]. These findings highlight that while behavioral interventions are important, addressing systemic social and economic factors is crucial to improving women’s health, providing additional evidence that broader SDOH gaps disproportionately impact women’s wellbeing [29, 30].

### Economic and structural challenges affecting women’s wellbeing

Females in the study also reported higher rates of transportation barriers (i.e. lack of available and reliable transport, such as a car or public transit), which can limit access to healthcare, employment, and other essential services. Women frequently shoulder unpaid caregiving responsibilities, earn less than men, and experience greater economic instability, all of which contribute to chronic stress and restrict access to healthcare, stable housing, and nutritious food [31, 32]. These compounded challenges reinforce the need for multi-level policies and community supports addressing economic inequities and caregiving burdens.

### Racial disparities within the female subgroup

Within female patients, racial and ethnic disparities were pronounced. American Indian or Alaska Native females reported the highest average number of affirmative SDOH responses (1.19 per person), indicating a substantial burden of adverse social conditions. They also experienced elevated rates of depression, stress, and food insecurity, reflecting compounded effects of economic and structural marginalization [33]. Research on Indigenous health remains limited, particularly regarding barriers to healthcare access such as insurance coverage and affordability. Moreover, state-recognized tribes lack access to Indian Health Service (IHS) resources, further restricting culturally appropriate care [34]. Existing literature tends to focus on individual-level factors rather than structural harms like colonization and economic disenfranchisement despite elevated morbidity and mortality rates [35].

Similarly, Black/African American females experienced greater financial strain and lower physical activity levels. These disparities stem from a complex interplay of intrapersonal (e.g., limited time, motivation), interpersonal (e.g., caregiving responsibilities), and environmental barriers (e.g., safety concerns, lack of facilities), underscoring the urgent need for culturally tailored wellness programs and policies that address racialized economic disparities [33, 36]. Addressing these inequities requires comprehensive, community-engaged solutions targeting systemic barriers disproportionately impacting women of color.

### Substance use and behavioral risks predominant among males

Conversely, males were more likely to report substance use, including higher rates of tobacco and alcohol use. White/Caucasian males showed the highest alcohol use prevalence (6.23%), and both White/Caucasian and Black/African American males exhibited the highest tobacco use rates (42%). Such behaviors are consistently linked to adverse long-term health outcomes and may reflect self-medication for untreated mental health issues [37]. Traditional gender norms discourage men from seeking mental health care, often resulting in untreated depression and anxiety that manifest as substance use, risk-taking, or externalizing behaviors [38]. This pattern aligns with prior findings suggesting men’s health risks are more behaviorally driven, whereas women’s health is more strongly influenced by structural determinants [19]. While behavioral interventions targeting male substance use remain essential, improving overall population health necessitates addressing the root socioeconomic causes of instability affecting women’s health.

### Limitations

This study has several limitations. First, the retrospective cross-sectional design precludes causal inference and reflects only a snapshot in time. Second, data were drawn from a single healthcare system in South Carolina, which may limit generalizability. However, South Carolina mirrors many national trends in health inequities, such as high rates of poverty, maternal morbidity, and chronic illness, particularly among women and racially marginalized groups [39, 40]. As such, while findings may not be universally representative, they likely reflect broader structural patterns relevant to similar healthcare contexts across the U.S. Third, the use of EHR data introduces risks of missing, incomplete, or inaccurate information. Most SDOH screenings were conducted in person by a provider, either during bedside assessments or clinic rooming processes, which may have discouraged full disclosure from patients, especially for sensitive topics like violence, housing instability, or substance use. This may have led to underreporting of key social needs. Fourth, all SDOH responses were self-reported, which introduces potential reporting bias, including both under- and over-reporting. Additionally, although sex was captured in the EHR, our ability to assess gender identity was limited, as standardized collection of gender identity data was only recently implemented. Future research should explore gender-based differences as this information becomes more widely available.

### Perspectives and significance

The significant sex differences in substance use, mental health, and social connectedness underscore the need for sex- and gender-responsive public health strategies to improve health outcomes and reduce disparities. For men, early behavioral interventions, such as programs targeting substance use initiation, increasing access to mental health services, and reducing stigma around help-seeking, can help mitigate long-term risks of chronic disease and poor mental health. For women, who often experience compounding social and economic disadvantages, interventions that go beyond individual behavior change are essential. These may include policies addressing financial insecurity, housing instability, and access to comprehensive healthcare, particularly for maternal and mental health, offering a more holistic, structural approach to improving well-being.

Importantly, systemic changes are critical for all patients, regardless of sex. However, our findings could suggest that women may benefit from more integrated, multifaceted interventions that combine clinical care with social support and economic policy reforms. For example, a comprehensive intervention for women might include coordinated care models that integrate primary, reproductive, and mental health services alongside access to housing assistance, wage equity initiatives, and trauma-informed peer support. For men, behavioral interventions may be particularly impactful when paired with structural supports such as accessible healthcare coverage and employment-based wellness programs focused on mental health and substance use prevention.

Addressing sex and gender disparities in health requires a broad, coordinated approach that targets both social systems and healthcare access. Structural interventions, such as wage equity, paid family leave, affordable childcare, and housing assistance, can promote economic stability and reduce stressors that disproportionately affect women [41]. Expanding Medicaid coverage and ensuring access to preventive and behavioral health services, including those specific to maternal mental health, are also critical strategies [42]. Embedding routine SDOH screening into healthcare systems, such as assessing for housing and food insecurity, IPV, mental health concerns, and transportation needs, could help identify and address social risk factors in a timely manner [43, 44].

While many sex differences in SDOH responses were statistically significant, some differences were small in magnitude and may reflect the large sample size rather than clinically meaningful variation. For instance, slightly higher rates of stress and financial strain among females align with broader population trends but may not necessitate sex-specific interventions in isolation. However, subgroup findings point to more urgent needs: American Indian or Alaska Native females reported the highest burden of SDOH risks, and White and Black males demonstrated disproportionately high rates of alcohol and tobacco use. These findings suggest that nuanced, intersectional approaches—accounting for both sex and race—are likely to be more effective than broad, population-wide strategies.

Overall, these results reinforce the need to integrate sex and gender considerations into clinical practice, public health planning, and policy development. Future research should examine how intersecting social identities, including race, socioeconomic status, and geographic location, further influence health disparities. Longitudinal studies could help identify how persistent structural barriers shape health trajectories over time, as well as which interventions, structural, behavioral, or both, yield the greatest impact in reducing gender- and race-based health inequities.

## Conclusions

Although males and females reported similar overall rates of SDOH needs, domain-specific differences revealed stark disparities in individual SDOH needs. Females faced heightened structural and social risks, whereas males are more likely to engage in behaviors that contribute to poorer overall health. Addressing these disparities requires a multidimensional approach that moves beyond individual behaviors to tackle systemic inequities. By integrating gender-specific considerations into public health and clinical care, more targeted, evidence-based interventions can be developed that directly address the distinct determinants of social drivers of health to promote equitable health outcomes.

## Data Availability

Data can be obtained from the corresponding author upon reasonable request.
